# CD4^+^ T cell count mediates the association between gut microbiota and diabetic kidney disease progression

**DOI:** 10.3389/fcimb.2026.1699989

**Published:** 2026-05-20

**Authors:** Xuejun Zheng, Yahan Zhu, Wen Cui, Zhihui Wang, Chenyan Xia, Yingxin Zhi, Jin Shang, Zhanzheng Zhao

**Affiliations:** 1 Department of Nephrology, The First Affiliated Hospital of Zhengzhou University, Zhengzhou, Henan, China; 2 Zhengzhou University, Zhengzhou, Henan, China; 3 Department of Nephrology, China-Japan Friendship Hospital, Chinese Academy of Medical Sciences & Peking Union Medical College, Beijing, China

**Keywords:** CD4^+^ T cell count, diabetic kidney disease, gut microbiota, gut-immune-kidney axis, mediation analysis

## Abstract

**Background:**

Diabetic kidney disease (DKD) is the main cause of end-stage renal disease (ESRD). Gut microbiota dysbiosis can affect the DKD progression through pathways involving metabolism and inflammation. However, the dynamic evolution of gut microbiota along various DKD stages, and its interaction with immune cells and the DKD exacerbation remain unclear. This study aimed to explore the characteristics of gut microbiota alterations during DKD progression and evaluate whether CD4^+^ T cell count showed a statistically significant mediating association in this process.

**Methods:**

A total of 157 patients with DKD were classified into early (n=34), middle (n=91) and late (n=32) stage groups according to clinical indicators. Fecal samples were collected for 16S rRNA sequencing to analyze the composition and diversity of the gut microbiota. Patients were further divided into low (n=22) and normal (n=44) CD4^+^ T cell count groups for comparative analysis. Mediation analysis was conducted to evaluate the relationship among gut microbiota, CD4^+^ T cells, and DKD progression.

**Results:**

The gut microbiota underwent significant changes at various stages of DKD. Thirty-five bacterial genera showed continuous changes in abundance during DKD progression. These bacterial genera were closely associated with renal function indicators and CD4^+^ T cell count. In addition, significant difference in the diversity and composition of gut microbiota were identified between the low and normal CD4^+^ T cell count groups, and were significantly related with renal injury markers. Mediation analysis showed that CD4^+^ T cell count mediated the association between 13 bacterial genera and the DKD progression.

**Conclusion:**

The gut microbiota undergoes dynamic evolution during DKD progression, with CD4^+^ T cell count showing a significant mediating association between gut microbiota and DKD progression. These findings support a potential gut–immune–kidney association framework and warrant further investigation.

## 1 Introduction

Diabetic kidney disease (DKD) is the most common microvascular complication of diabetes mellitus (DM) and the main cause of end-stage renal disease (ESRD) (Wang et al., 2023). The latest data from the International Diabetes Federation (IDF) indicates that the number of adults aged 20–79 with diabetes worldwide reached 589 million in 2024, accounting for 11.1% of the total population in this age group. In individuals aged 65 years and older, 25% is affected by DKD. DKD not only seriously endangers patients’ life, health and quality of life, but also imposes a huge socioeconomic burden (Gülümsek and Keşkek, 2022). The DKD pathogenesis is influenced by multiple factors, among which metabolism and inflammation play key roles (Qin et al., 2025). However, the specific mechanism underlying the DKD progression remains unclear (Lassén and Daehn, 2020). Therefore, it is urgently necessary to explore the mechanisms underlying the occurrence and development of DKD, so as to identify directions for its prevention and treatment.

A growing body of evidence demonstrates that the gut microbiota is closely associated with various kidney diseases (Myagmankhuu et al., 2025; Hua et al., 2026; Sun et al., 2026). The normal structure of the gut microbiota plays an important role in maintaining the body’s homeostasis (Jandhyala, 2015). Intestinal dysbiosis can affect the occurrence and development of kidney diseases through multiple pathways such as metabolism and inflammation. Disturbed gut microbiota is involved in the onset of membranous nephropathy (MN), which can be used for non-invasive diagnosis (Shang et al., 2022b). Previous studies have shown that compared with non-DKD populations (including diabetic patients and healthy controls), DKD patients exhibit significant differences in gut microbiota characteristics, which affects the DKD progression through metabolic pathways (Shang et al., 2022a). However, the dynamic evolution of gut microbiota across DKD stages remains poorly understood.

Renal inflammatory response also plays an indispensable role in the DKD progression (Tang and Yiu, 2020). The release of inflammatory mediators facilitates the infiltration of immune cells into the kidneys (Rayego-Mateos et al., 2023). Previous studies have found that the high-density infiltration of CD4^+^ T cells is closely related to the severity of renal injury (Han et al., 2024). The infiltration of CD4^+^ T cells in the kidneys promotes the occurrence of nephritis in MRL/lpr mice (Okamoto et al., 2012). CD4^+^ T cells also promote renal fibrosis by up-regulating the expression of type I collagen (Tapmeier et al., 2010). The gut microbiota is also closely associated with the immune system. Butyrate, a metabolite of intestinal microorganisms, affects the differentiation of CD4^+^ T cells and balances the ratio of Th17 (T helper 17) to Treg (regulatory T) cells (Xing et al., 2025). However, the relationship among the gut microbiota, immune cells, and the DKD progression has not yet been clarified.

This study aimed to explore the characteristics of continuous changes in the gut microbiota during different stages of DKD progression, and to investigate the potential hub role of CD4^+^ T cells in this process. We attempted to construct a mediation model, among “gut microbiota–CD4^+^ T cells–DKD progression”, which may provide novel therapeutic strategy for DKD intervention.

## 2 Design and methods

### 2.1 Participant identification and sample collection

The clinical information and fecal samples from 157 patients with DKD were collected, who were admitted to the First Affiliated Hospital of Zhengzhou University from October 2018 to October 2019. All patients included in this project provided written informed consents. The inclusion criteria were as follows: 1) met the criteria for diabetes diagnosis, 2) urinary albumin-to-creatinine ratio (UACR) ≥30 mg/g and/or estimated glomerular filtration rate (eGFR) <60 mL/min, sustained for more than 3 months. The exclusion criteria were as follows: 1) acute diabetic complications, such as diabetic ketoacidosis or hyperosmolar hyperglycemic state, 2) severe infections, severe dysfunction of the heart, liver, or kidneys, hematologic diseases, malignancies, pregnancy, or other such conditions, 3) primary glomerulonephritis diagnosed by renal biopsy, 4) untreated urinary tract infections or diseases affecting urinary protein levels, 5) missing key data or abnormal values. Peripheral blood lymphocyte subsets were determined using an Agilent NovoCyte D2060R flow cytometer at the hospital’s clinical immunology laboratory. Analysis was performed using NovoExpress software (Agilent Technologies Inc., USA). Peripheral blood mononuclear cells were stained with the following antibodies: anti-CD45 (PerCP), anti-CD3 (FITC), anti-CD4 (APC), anti-CD8 (PE), anti-CD19 (APC), and anti-CD56 (PE). Absolute lymphocyte subset counts were obtained volumetrically using the instrument’s high-precision injection pump according to the manufacturer’s instructions. The included patients were classified into three groups according to KDIGO guidelines and clinical diagnostic criteria: early stage (microalbuminuria): UACR 30–300 mg/g, or 24-hour urinary albumin excretion (24h UAE) 30–300 mg/24h, with eGFR >15 mL/min/1.73 m²; middle stage (albuminuria): UACR >300 mg/g, or 24h UAE >300 mg/24h, with eGFR >15 mL/min/1.73 m²; and late stage (uremia/ESRD): eGFR <15 mL/min/1.73 m², irrespective of albuminuria level. For further research, we divided DKD patients into low CD4^+^ T cell count group (L_CD4^+^ T, <550/μL) and normal CD4^+^ T cell count group (N_CD4^+^ T, 550-1440/μL) based on CD4^+^ T cell count. Propensity score matching was then performed at a 1:2 ratio, with adjustments made for potential confounding factors, including demographic and clinical indicators such as age, gender, and blood lipids.

### 2.2 DNA extraction, 16S rRNA amplification, and gene sequencing

Fecal samples from the 157 included patients were collected using a disposable fecal collection device and stored at -80 °C within 2 hours for further analysis. As previously described, we extracted microbial genomic DNA from fecal samples (Coll et al., 1989). After testing the DNA quality, we amplified the DNA fragments encoding the V3-V4 region of 16S ribosomal RNA (rRNA) in each sample using polymerase chain reaction (PCR) (Tiveljung et al., 1995). All fecal samples were suspended in 790μl of sterile lysis buffer (4M guanidine thiocyanate, 250ul; 10% N-lauroyl sarcosine, 40ul; 5% N-lauroyl sarcosine-0.1 M phosphate buffer [pH 8.0], 500ul) in 2 ml screw-cap tube containing 1g glass beads (0.1mm BioSpec Products, Inc., USA). This mixture was vortexed vigorously then incubate at 70°C for 1h. After incubation, bead beating was performed for 10min at maximum speed to fully disrupt cell membranes and nuclear membranes. We extracted DNA using the E.Z.N.A. Stool DNA Kit (Omega Bio-tek, Inc., GA, USA) and stored it at -20 °C for further analysis (Yin et al., 2016). Primers F1 and R2 (5′-CCTACGGGNGGCWGCAG-3′ and 5′-GACTACHVGGGTATCTAATCC-3′) correspond to positions 341–805 in the 16S rRNA gene of *Escherichia coli*, and were employed to amplify the V3–V4 region via PCR. We ran PCRs in an EasyCycler 96 PCR system (Analytik Jena Corp., AG) using the following program: 3 minutes at 95 °C followed by 21 cycles of 0.5 min at 94 °C (denaturation), 0.5 min at 58 °C for annealing, and 0.5 minutes at 72 °C (elongation), with a final extension at 72 °C for 5 min. 16S rRNA gene sequencing was conducted by Shanghai Biomedical Technology Co. Ltd. using the NextSeq 2000 platform (Illumina, USA). To reduce the confounding effects caused by technical methods, nucleotide sequencing of all samples was performed in one single sequencing center with synchronized standard operating procedures (Zhang et al., 2022).

### 2.3 Bioinformatic analysis

We used the DADA2 algorithm to denoise the raw sequencing data and obtain the amplicon sequence variant (ASV) table (Narayan et al., 2020). The representative sequences of each ASV were annotated based on the SILVA reference database (SSU138.2) to obtain information on species composition in the samples through taxonomic analysis. To minimize differences in sequencing depth among samples, the original ASV abundance matrix was subsampled 100 times at a sequencing depth of 10, 000 to generate a rarefied ASV subset for subsequent diversity analysis. Alpha diversity analysis was conducted to estimate the bacterial richness and evenness, including the ACE index, Chao index, Shannon index, and Simpson index (Bai et al., 2017). Principal coordinates analysis (PCoA) was used to compare the differences in community structure among groups, based on Bray-Curtis, unweighted, and weighted UniFrac algorithm (Xiong et al., 2015). Permutational multivariate analysis of variance (PERMANOVA) was performed to statistically test the differences in overall microbiota among groups (De Steenhuijsen Piters et al., 2016). We used R software to draw bar charts of species composition in each sample from the phylum to genus levels. We analyzed the differences in relative abundance of bacterial taxa among groups. Differences between two groups were analyzed using the nonparametric Mann-Whitney U test, and differences among three or more groups were analyzed using the Kruskal-Wallis rank-sum test. To reduce false-positive findings due to multiple comparisons, the resulting p-values were adjusted using false discovery rate (FDR) correction, with an adjusted p-value < 0.05 indicating significant differences. Because these analyses were performed on relative abundance data, the findings were interpreted cautiously and regarded as exploratory rather than definitive compositional differential abundance results. We used the linear discriminant analysis (LDA) effect size (LEfSe, version 1.1, https://github.com/SegataLab/lefse) to detect taxa with differential abundance among groups (Xiao C et al., 2016). We used a random forest model classifier model to identify key ASVs with significant differences between groups at the ASV level (Xiao et al., 2016) and generated an abundance heatmap. We performed functional gene and metabolic pathway prediction for the microbiota using the Phylogenetic Investigation of Communities by Reconstruction of Unobserved States (PICRUSt2) (https://github.com/picrust/picrust2/wiki) (Douglas et al., 2020). We identified differentially expressed metabolic pathways between groups via LEfSe analysis. The abundance data of the microbiota in the samples were correlated with physiological indicator data using Spearman correlation analysis, and a correlation heatmap was drawn to illustrate the relationships between the microbiota and important physiological indicators.

### 2.4 Mediation analysis

To determine whether the association between bacterial genera exhibiting continuous changes across DKD stages and DKD progression is mediated by CD4^+^ T cell count, a mediation analysis was conducted. The bacterial genera abundance and DKD stages were designated as the independent variable (X) and the dependent variable (Y), respectively, with CD4^+^ T cell count as the mediator (M). In the mediation model, the hypothetical path was X→M→Y, and two paths needed to be verified: (1) the effect of X on M (X → M), and (2) the effect of M on Y after controlling for X (M → Y|X). Path 1 (X → M) was analyzed using linear regression (lm) (Li et al., 2023). Path 2 (M → Y|X) was analyzed using ordered logistic regression (polr/clm). The abundance of bacterial genera was normalized and then log-transformed to achieve standardization, and CD4^+^ T cell count underwent log transformation. A structural equation model (SEM) was constructed using the lavaan package in R software to analyze the indirect effect of bacterial genera on DKD stages through CD4^+^ T cell count (Average Causal Mediation Effect, ACME), the direct effect of bacterial genera on DKD stages (after controlling for CD4^+^ T cell count) (Average Direct Effect, ADE), and the proportion of the indirect effect to the total effect (Prop. Mediated). A significant mediation effect was confirmed if the ACME exhibited a p-value <0.05 and a 95% confidence interval excluding zero.

### 2.5 Statistical analysis

When describing clinical data, continuous variables were presented as mean ± standard deviation (SD) if normally distributed, or as median and interquartile range (IQR) if non-normally distributed. Categorical variables were expressed as absolute frequencies and percentages. When comparing two groups, Student’s t-test was applied to normally distributed data, the Mann-Whitney U test to non-normally distributed data, and the chi-square test or Fisher’s exact test to categorical variables. For multiple groups comparisons, one-way analysis of variance (one-way ANOVA) was used for normally distributed data, the Kruskal-Wallis test for non-normally distributed data, and the Chi-square test or Fisher’s exact test for categorical variables. A p-value < 0.05 was considered statistically significant.

## 3 Results

### 3.1 Baseline characteristics of participants

**Figure 1:**
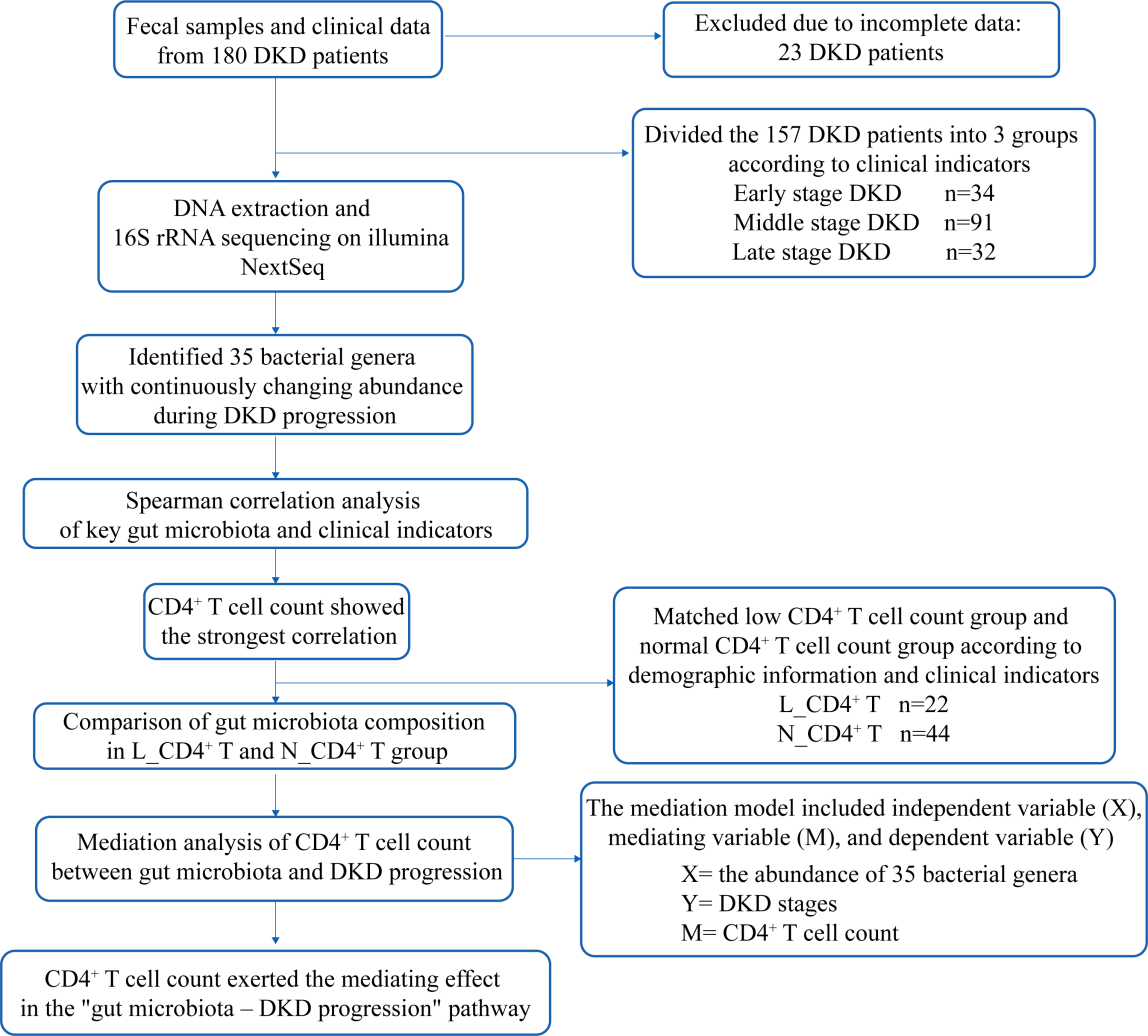
Study flowchart. DKD, diabetic kidney disease;N_CD4^+^ T, normal CD4^+^ T cell count; L_CD4^+^ T, low CD4^+^ T cell count. Matching indicators include age, gender, systolic blood pressure, diastolic blood pressure, Hemoglobin A1c, glucose, serum albumin, total cholesterol, triglycerides, history of glucocorticoids and immunosuppressant medication.

In this project, fecal samples of 180 DKD patients were collected. Due to missing clinical data from 23 participants, 157 patients were ultimately included in the analysis. Based on clinical indicators, these patients were divided into three groups: the Early stage group (Early, n = 34), Middle stage group (Middle, n = 91) and Late stage group (Late, n = 32). The study flowchart is shown in **Figure 1**. As shown in **Table 1**, significant differences were observed in serum creatinine (SCr), eGFR, serum albumin (ALB), UACR, 24h UAE among the three groups (p <0.001). Notably, differences were also found in immunological parameters including absolute count of lymphocyte (Lym#), T lymphocyte (T#), CD4^+^ T lymphocyte (CD4^+^ T#), and B lymphocyte (B#) (p < 0.05), while no significant statistical differences in the status of taking glucocorticoids and immunosuppressant (p > 0.05).

**Table 1:** Baseline characteristics of participants stratified by DKD stage.

Clinical indicators	Total (n = 157)	Early stage_DKD (n = 34)	Middle stage_DKD (n = 91)	Late stage_DKD (n = 32)	p-value
Age (years)	55.00 (49.00, 62.00)	54.00 (50.25, 60.75)	55.00 (49.00, 63.00)	57.00 (52.00, 59.25)	0.686
Gender					0.996
female	59 (37.58)	13 (38.24)	34 (37.36)	12 (37.5)	
male	98 (62.42)	21 (61.76)	57 (62.64)	20 (62.50)	
SBP (mmHg)	138.00 (126.00, 150.00)	130.00 (122.00, 140.00)	140.00 (127.25, 150.00)	144.00 (132.00, 165.00)	0.003
DBP (mmHg)	80.97 ± 11.14	79.24 ± 9.73	80.28 ± 10.53	84.97 ± 13.57	0.081
DM course(months)	120.00 (48.00, 180.00)	72.00 (24.00, 156.00)	114.00 (48.00, 159.00)	168.00 (105.00, 240.00)	0.008
HbA1c (%)	7.32 (6.60, 8.74)	7.04 (6.63, 8.51)	7.67 (6.74, 8.98)	6.78 (6.07, 8.12)	0.029
Glu (mmol/L)	5.39 (4.42, 7.01)	5.35 (4.94, 7.71)	5.40 (4.17, 7.17)	5.26 (4.38, 6.65)	0.546
SCr (μmol/L)	120.50 (74.00, 258.52)	100.80 (64.00, 118.00)	107.00 (72.05, 166.30)	514.25 (472.70, 765.00)	<.001
eGFR (mL/min/1.73 m²)	52.43 (21.39, 81.95)	65.41 (49.07, 102.19)	62.21 (33.04, 94.33)	7.99 (6.03, 10.34)	<.001
ALB (g/L)	35.10 (27.95, 39.50)	41.30 (35.95, 42.85)	31.10 (26.55, 37.35)	36.00 (31.57, 39.42)	<.001
TCHO (mmol/L)	4.53 (3.61, 5.63)	4.22 (3.31, 4.73)	4.81 (3.74, 6.13)	4.23 (3.62, 5.14)	0.006
TG (mmol/L)	1.56 (1.15, 2.38)	1.56 (1.14, 2.02)	1.58 (1.20, 2.51)	1.47 (0.93, 2.37)	0.421
UACR (mg/g)	1510.50 (425.68, 3130.85)	101.46 (62.11, 171.29)	1974.03 (784.36, 3348.05)	2981.17 (1586.08, 4398.32)	<.001
24h UAE (mg/24h)	1255.30 (321.00, 3503.52)	131.04 (77.79, 252.16)	2245.32 (917.50, 4200.42)	2726.16 (1355.96, 3619.44)	<.001
Lym# (cells/μL)	1638.00 (1200.00, 2325.00)	1847.00 (1421.00, 2425.50)	1773.00 (1324.50, 2423.50)	1091.00 (941.00, 1374.00)	0.003
T# (cells/μL)	1197.00 (870.50, 1641.50)	1492.00 (1035.00, 1681.00)	1223.00 (977.00, 1751.00)	852.00 (693.50, 1053.00)	0.003
CD4^+^ T# (cells/μL)	716.50 (490.75, 1047.50)	782.50 (552.50, 1071.75)	785.00 (554.00, 1069.00)	480.00 (420.50, 590.50)	0.004
CD8^+^ T# (cells/μL)	427.00 (279.00, 610.00)	465.50 (261.75, 637.50)	438.00 (311.00, 610.00)	357.00 (228.75, 437.75)	0.321
B# (cells/μL)	209.50 (146.50, 339.75)	217.00 (174.00, 331.00)	228.00 (148.75, 374.75)	109.00 (70.00, 166.00)	0.003
NK# (cells/μL)	143.00 (88.25, 275.25)	152.00 (100.00, 297.50)	168.00 (87.00, 275.25)	95.00 (57.00, 135.00)	0.084
Glucocorticoids					0.089
No	137 (87.26)	27 (79.41)	79 (86.81)	31 (96.88)	
Yes	20 (12.74)	7 (20.59)	12 (13.19)	1 (3.12)	
Immunosuppressant					0.506
No	126 (80.25)	27 (79.41)	71 (78.02)	28 (87.50)	
Yes	31 (19.75)	7 (20.59)	20 (21.98)	4 (12.50)	

DKD, diabetic kidney disease; SBP, systolic blood pressure; DBP, diastolic blood pressure; DM course, course of diabetes mellitus; HbA1c, Hemoglobin A1c; Glu, blood glucose; SCr, serum creatinine; eGFR, estimated glomerular filtration rate; ALB, serum albumin; TCHO, total cholesterol; TG, triglyceride; UACR, urine albumin-to-creatinine ratio; 24h UAE, 24-hour urinary albumin excretion; #, absolute count; Lym, lymphocyte; T, T lymphocyte; CD4^+^ T, CD4^+^ T lymphocyte; CD8^+^ T, CD8^+^ T lymphocyte; B, B lymphocyte; NK, NK cell.

### 3.2 Significant gut microbiota alterations occurred during DKD progression

**Figure 2:**
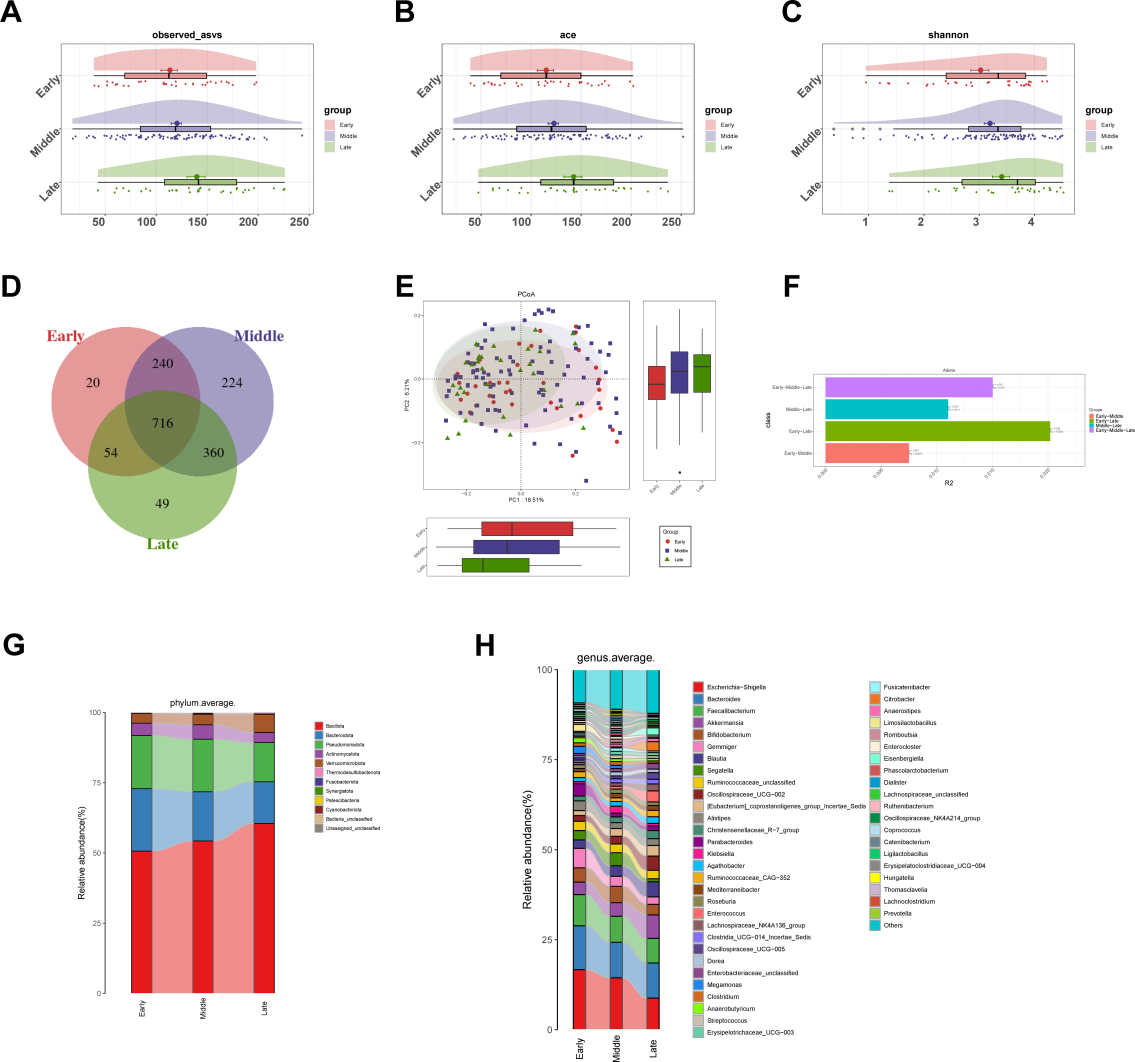
Comparison of gut microbiota diversity and composition in DKD stages. **(A–C)** The comparison of gut microbiota richness and diversity among early (n=34), middle (n=91) and late (n=32) DKD stages was presented through observed amplicon sequence variants (ASVs) (p = 0.035), ace index (p = 0.034) and Shannon index (p = 0.048). **(D)** Venn diagram showed the number of shared and unique ASVs among the three groups. **(E)** Based on unweighted Unifrac distances, the differences in microbial community structure were displayed through principal co-ordinates analysis (PCoA). **(F)** Permutational multivariate analysis of variance (PERMANOVA) showed significant differences in gut microbiota composition among the three DKD stages (R^2^ = 0.015, p = 0.033). **(G, H)** Comparison of gut microbiota abundance among the three groups at the phylum and genus level. Statistical analysis: Panels **(A–C)** were analyzed using the Kruskal–Wallis test, and panel F shows the PERMANOVA result. Early, early stage DKD; Middle, middle stage DKD; Late, late stage DKD.

We analyzed the microbiota diversity of the Early stage, Middle stage and Late stage groups. With the progression of DKD stages, alpha-diversity indices reflecting richness and evenness showed significant intergroup differences, including observed ASVs (p = 0.035), ACE (p = 0.034), Shannon (p = 0.048), and Chao (p = 0.035) (**Figures 2A–C**; **Supplementary Figure S1A**). The Venn diagrams illustrated the similarity and overlap of ASVs among the three groups (**Figure 2D**). The PCoA and showed that the community composition of three groups was dramatically separated (**Figure 2E**), which was support by PERMANOVA (R^2^ = 0.015, p = 0.033) (**Figure 2F**), while non-metric multidimensional scaling (NMDS) analysis and constrained principal coordinates analysis (CPCoA) showed similar results (**Supplementary Figures S1B, C**). LEfSe was used to filter phylogenetic composition and comparison from phylum to genus level of the gut microbiota in the three groups (**Supplementary Figures S1D, E**). The average gut microbiota in three groups was dominated by phyla *Bacillota, Bacteroidota, Pseudomonadota, Actinomycetota* and *Verrucomicrobiota* with the sum totals more than 95% in these groups (**Figure 2G**). At the genus level, *Escherichia-Shigella* was significantly decreased during the gradual progression of DKD stages, followed by *Bacteroides, Faecalibacterium, Akkermansia* and *Blautia*. However, *Blautia* showed a considerable increase with the gradual progression of DKD stages, followed by *Oscillospiraceae_UCG-002, [Eubacterium]_coprostanoligenes_group_Incertae_Sedis, Christensenellaceae_R-7_group* and *Agathobacter* (**Figure 2H**). At the phylum (**Supplementary Figure S1F**) and genus (**Supplementary Figure S1G**) levels, the abundance of gut microbiota showed differences in three groups.

### 3.3 The bacterial genera with continuously changing abundance during DKD progression were significantly correlated with CD4^+^ T cells

**Figure 3:**
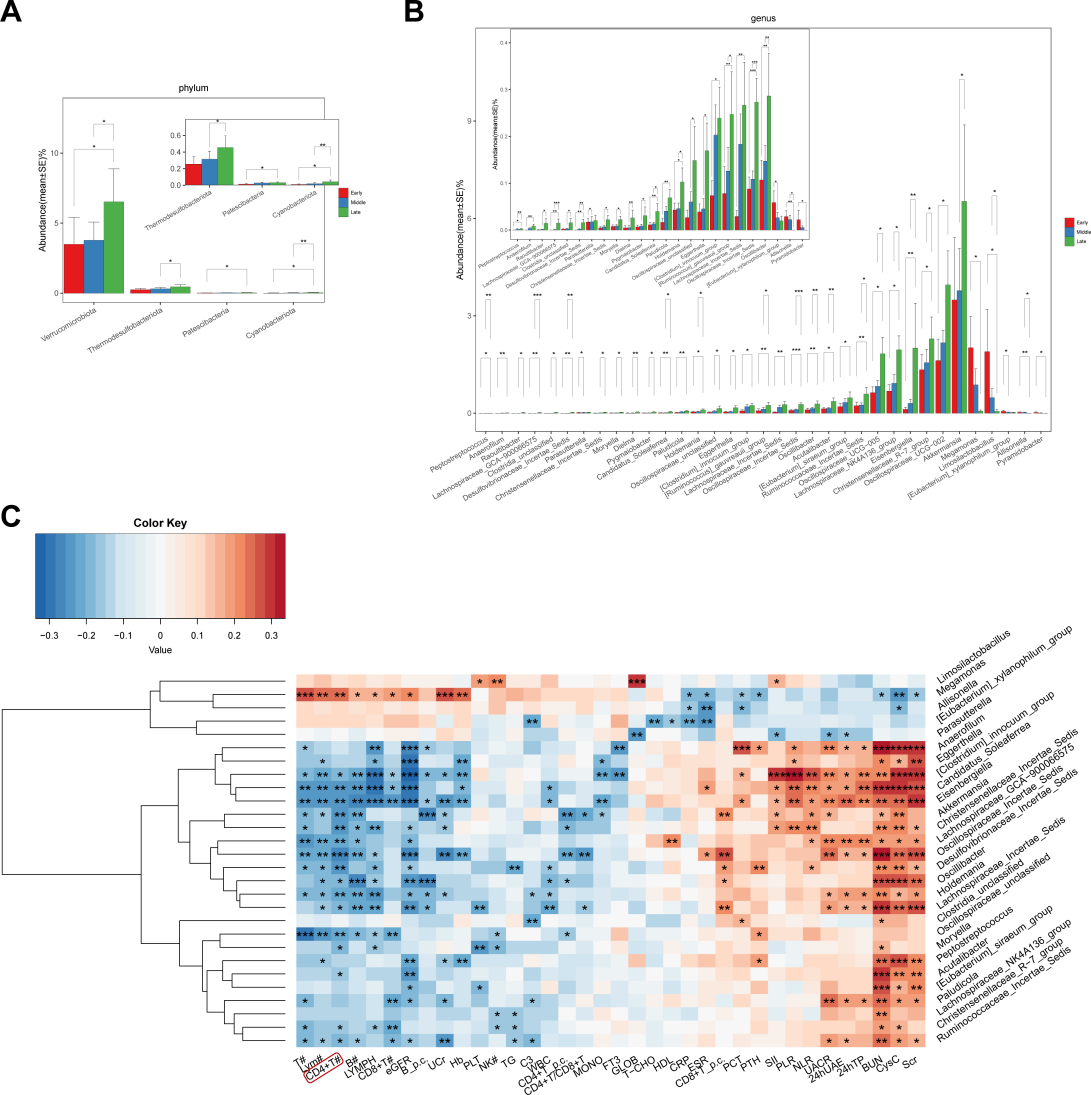
Microbiota exhibiting continuous abundance changes during DKD progression and Spearman correlation analysis with clinical indicators. **(A, B)** The relative abundance of gut microbiota at the phylum and genus level that exhibit progressive changes across DKD stages. **(C)** Spearman correlation analysis showed the correlation between key bacterial genera and clinical indicators. *p < 0.05, **p < 0.01, ***p < 0.001. T#, absolute account of total T lymphocytes; Lym#, absolute account of lymphocytes; CD4^+^ T#, absolute account of CD4^+^ T cells; B#, absolute account of B lymphocytes; LYMPH, lymphocyte; CD8^+^ T#, absolute account of CD8^+^ T cells; eGFR, estimated glomerular filtration rate; B_p.c., percentage of B cells; UCr, urinary creatinine; Hb, hemoglobin; PLT, platelet; NK#, natural killer cell absolute count; TG, triglyceride; C3, C3 complement; WBC, white blood cell; CD4^+^ T_p.c., percentage of CD4^+^ T cells; CD4^+^ T/CD8^+^ T, CD4^+^ T cell count/CD8^+^ T cell count; MONO, monocyte; FT3, free triiodothyronine; GLOB, globulin; T-CHO, total cholesterol; HDL, high-density lipoprotein; CRP, C-reactive protein; ESR, erythrocyte sedimentation rate; CD8^+^ T_p.c., percentage of CD8^+^ T cells; PCT, procalcitonin; PTH, parathyroid hormone; SII, systemic immune-inflammation index; PLR, peripheral blood platelet count/lymphocyte count; NLR, peripheral blood neutrophil count/lymphocyte count; UACR, urinary albumin-to-creatinine ratio; 24h UAE, 24-hour urinary albumin excretion rate; 24hTP, 24-hour urine total protein; BUN, blood urea nitrogen; CysC, cystatin C; Scr, serum creatinine.

We screened out the microbiota whose abundance showed continuous increasing or decreasing during the progression of DKD stages. At the phylum level, the abundance of four bacterial phyla which were *Verrucomicrobiota*, *Thermodesulfobacteriota, Patescibacteria*, and *Cyanobacteriota* exhibited a continuous increase with the progression of DKD stages (**Figure 3A**). From class to family level, there were also some gut microbiota whose abundance changes dynamically with the progression of DKD stages, such as *Negativicutes*, *Lachnospirales* and *Lachnospiraceea* (**Supplementary Figures S2A–C**). At the genus level, a total of 35 bacterial genera were found to show a continuous changing trend in their abundances across the stages of DKD. Among them, the abundances of 30 genera, such as *Oscillibacter, Eggerthella* and *Oscillospiraceae_UCG-002*, gradually increased during the progression of DKD stages; while the abundances of 5 genera, including *Megamonas* and *Limosilactobacillus*, continuously decreased (**Figure 3B**). To further explore their function, Spearman correlation analysis was performed to determine the relationship between the screened bacterial genera with continuous changes with the physiological indices of DKD patients. It was found that these bacterial genera were significantly associated with renal function-related indicators, such as eGFR, SCr, UACR, 24h UAE, etc. In addition, CD4^+^ T cell count also showed significant correlations, with the direction of correlation consistent with that of eGFR, showing a negative correlation with the abundance of the vast majority of bacterial genera (**Figure 3C**). Similarly, in the Spearman correlation analysis between key ASVs and clinical indicators, CD4^+^ T cell count also demonstrated significant correlations (**Supplementary Figure S2D**).

### 3.4 The structure of the gut microbiota varied across groups with different CD4^+^ T cell counts

**Figure 4:**
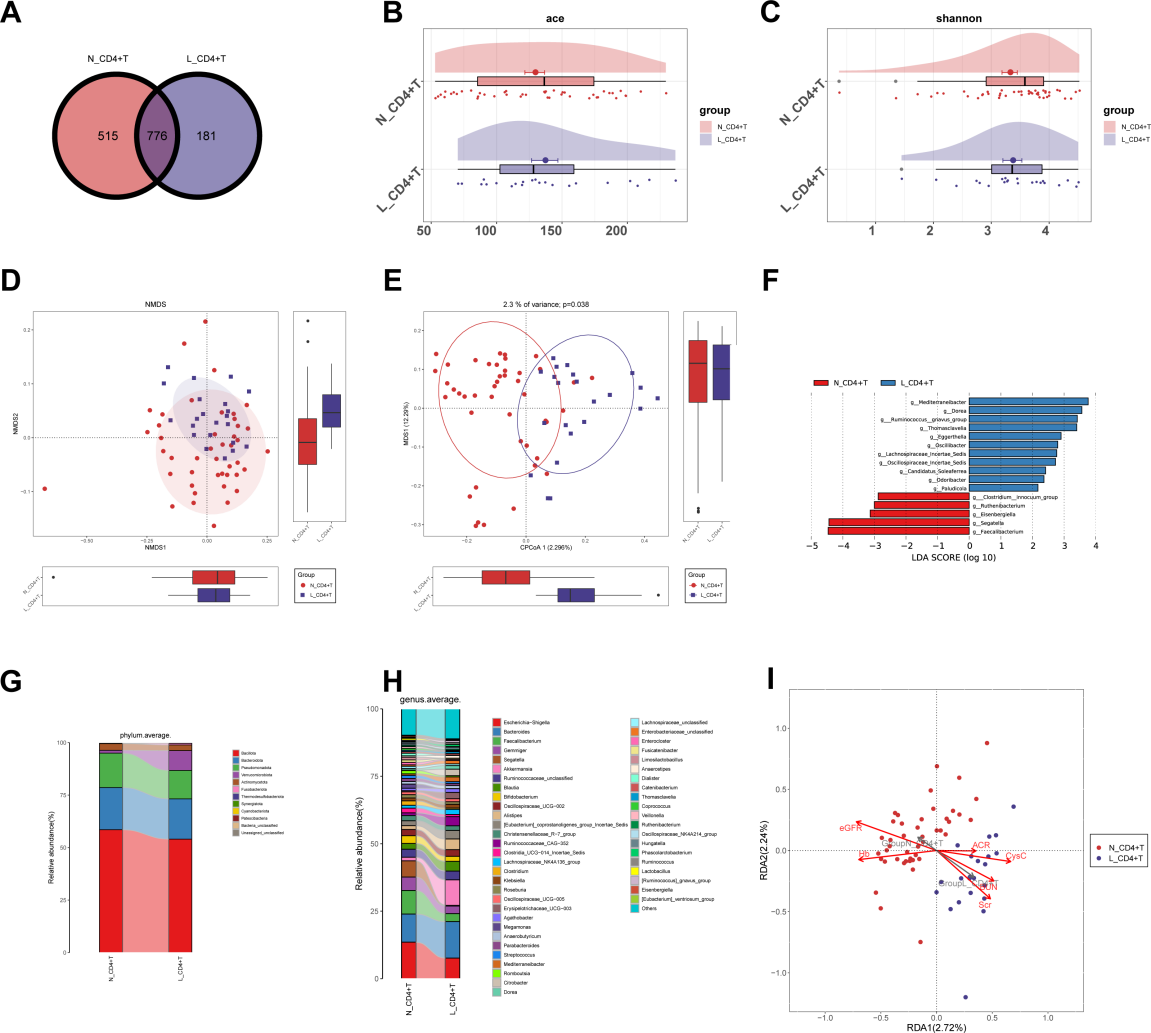
Comparison of gut microbiota diversity and composition in N_CD4^+^ T group and L_CD4^+^ T group. **(A)** Venn diagram showed the distribution of ASVs in N_CD4^+^ T (n=44) and L_CD4^+^ T (n=22) groups. **(B, C)** Cloudplot showed α-diversity in N_CD4^+^ T and L_CD4^+^ T by ace index (p = 0.056) and Shannon index (p = 0.096). **(D, E)** Non-metric multidimensional scaling (NMDS) and canonical analysis of principal coordinates (CAP) showed the gut microbiota and inter-group variations between the two groups. **(F)** Linear discriminant analysis (LDA) effect size (LEfSe) analysis of microbial profiles at the genus level between the N_CD4^+^ T group and the L_CD4^+^ T group. **(G, H)** Comparison of gut microbiota abundance among the two groups at phylum and genus levels. **(I)** Redundancy analysis (RDA) was performed to investigate the correlation between gut microbiota of the two groups and clinical indicators. Statistical analysis: Panels **(B, C)** were analyzed using the Mann–Whitney U test, and panels **(D, E, I)** present ordination analyses for visualization. N_CD4^+^ T, normal CD4^+^ T cell count; L_CD4^+^ T, low CD4^+^ T cell count.

In view of the significant correlation between CD4^+^ T cell count and specific continuously changing genera during DKD progression, we reclassified patients based on CD4^+^ T cell count. We selected the patients with CD4^+^ T cell below the normal range (L_CD4^+^ T, <550/μL) and within the normal range (N_CD4^+^ T, 550-1440/μL). After propensity score matching, 22 and 44 patients were included respectively (**Table 2**). We found a total of 1291 ASVs in the N_CD4^+^ T group and 957 in the L_CD4^+^ T group. And 776 of the total 1, 472 ASVs were shared by both groups (**Figure 4A**). As indicated by the alpha diversity index, no significant differences in richness or diversity were observed between the N_CD4^+^ T group and the L_CD4^+^ T group, including ACE (p = 0.056), Shannon (p = 0.096), and Chao (p = 0.055) (**Figures 4B, C**; **Supplementary Figure S3A**). We used the NMDS analysis and CPCoA to reveal the significant differences between the community composition of the two groups (**Figures 4D, E**), which was consistent with the results of PCoA and PLSDA (**Supplementary Figures S4C, D**). Using LEfSe analysis, we identified gut microbiota with significant differences in abundance at the genus level (**Figure 4F**), and their phylogenetic distribution from phylum to genus level was illustrated in the cladogram (**Supplementary Figure S3B**). Analysis of intergroup differential species showed that, at the phylum level, the *Bacillota* was significantly accumulated in the N_CD4^+^ T group compared with the L_CD4^+^ T group. However, *Verrucomicrobiota* and *Fusobacteriota* were significantly reduced in the L_CD4^+^ T group (**Figure 4G**). At the genus level, the abundance of *Escherichia-Shigella* was significantly increased in the N_CD4^+^ T group, followed by *Faecalibacterium* and *Gemmiger*. Nevertheless, compared with the L_CD4^+^ T group, the abundance of *Bacteroides, Akkermansia* and *Blautia* were lower in N_CD4^+^ T group (**Figure 4H**). At the phylum (**Supplementary Figure S3E**) and genus (**Supplementary Figure S3F**) levels, the abundance of gut microbiota showed significant differences using Mann–Whitney U test. Redundancy analysis (RDA) revealed significant differences in gut microbiota composition among CD4^+^T cell subgroups, which correlated well with renal function-related indicators. The L_CD4^+^ T group was highly associated with renal injury markers (elevated blood urea nitrogen (BUN), Scr, (cystatin C) CysC, and UACR), and negatively correlated with renal protective indicators (higher eGFR and (hemoglobin) Hb) (**Figure 4I**). These results further confirmed that low CD4^+^ T cell count is correlated with the promotion of DKD progression by gut microbiota.

**Table 2:** Population characteristics of 1:2 matching between low CD4^+^ T cell count and normal CD4^+^ T cell count group.

Clinical indicators	Total (n = 66)	L_CD4^+^T (n = 22)	N_CD4^+^T (n = 44)	p-value
Age (years)	56.33 ± 11.26	59.09 ± 9.35	54.95 ± 11.97	0.161
Gender				0.476
female	26 (39.39)	10 (45.45)	16 (36.36)	
male	40 (60.61)	12 (54.55)	28 (63.64)	
SBP (mmHg)	138.50 (127.00, 151.50)	142.00 (130.25, 152.75)	136.00 (125.50, 150.00)	0.483
DBP (mmHg)	80.00 (76.00, 86.75)	80.00 (76.25, 87.75)	79.00 (75.00, 86.00)	0.553
HbA1c (%)	7.08 (6.67, 8.59)	7.08 (6.62, 8.59)	7.08 (6.69, 8.49)	0.812
Glu (mmol/L)	5.39 (4.62, 6.85)	5.37 (4.56, 6.81)	5.39 (4.84, 6.79)	0.812
ALB (g/L)	34.56 ± 7.34	35.07 ± 8.00	34.31 ± 7.07	0.696
TCHO (mmol/L)	4.52 ± 1.27	4.60 ± 1.45	4.48 ± 1.19	0.723
TG (mmol/L)	1.43 (1.10, 1.94)	1.43 (1.16, 1.77)	1.42 (1.10, 1.96)	0.865
CD4^+^ T# (cells/μL)	650.00 (481.00, 967.75)	443.50 (393.00, 479.50)	846.00 (653.00, 1061.50)	<.001
Glucocorticoids				0.129
No	55 (83.33)	21 (95.45)	34 (77.27)	
Yes	11 (16.67)	1 (4.55)	10 (22.73)	
Immunosuppressant				0.320
No	49 (74.24)	18 (81.82)	31 (70.45)	
Yes	17 (25.76)	4 (18.18)	13 (29.55)	

L_CD4^+^ T, low CD4^+^ T cell count; N_CD4^+^ T, normal CD4^+^ T cell count; SBP, systolic blood pressure; DBP, diastolic blood pressure; HbA1c, Hemoglobin A1c; Glu, blood glucose; ALB, serum albumin; TCHO, total cholesterol; TG, triglyceride; CD4**^+^**T#, absolute count of CD4**^+^** T lymphocyte.

### 3.5 CD4^+^ T cell count mediated the association between gut microbiota and DKD progression

**Figure 5:**
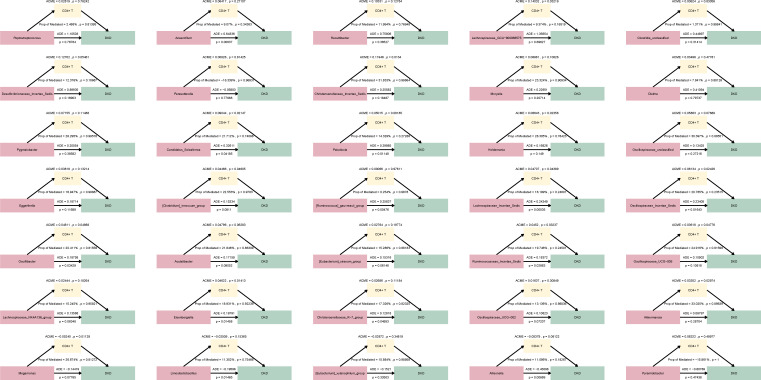
Mediation analysis of CD4^+^ T cell count between gut microbiota with continuous abundance changes during DKD stages and DKD progression. ACME, average causal mediation effect; ADE, average direct effect; Prop of Mediated, proportion of the mediated effect to the total effect.

To explore whether CD4^+^ T cell count showed a mediating association between the 35 genera with changing abundances and DKD progression, we constructed a mediation model among “gut microbiota–CD4^+^ T cells–DKD progression”. If the ACME exhibited a p-value <0.05 and the 95% confidence interval (95%CI) excluded zero, it indicated that the mediating effect existed (Göktepe et al., 2025). By comparison, we found 13 out of the 35 genera indicated mediating effect, including *Lachnospiraceae_GCA-900066575*, *Christensenellaceae_Incertae_Sedis*, *Candidatus_Soleaferrea*, *Holdemania*, *[Clostridium]_innocuum_group*, *Lachnospiraceae_Incertae_Sedis*, *Oscillospiraceae_Incertae_Sedis*, *Oscillibacter*, *Ruminococcaceae_Incertae_Sedis*, *Oscillospiraceae_UCG-005*, *Eisenbergiella*, *Akkermansia* and *Megamonas* (**Figure 5**). This supported a statistically significant mediating association of CD4^+^ T cell count in the relationship between specific gut microbiota and DKD progression.

## 4 Discussion

The results of this study indicated that during the progression of DKD stages, the abundance of 35 bacterial genera underwent significant and continuous changes. Moreover, CD4^+^ T cells were closely associated with the aforementioned gut microbiota and showed a significant mediating association within the relationship between gut microbiota and DKD progression.

The composition of gut microbiota changed significantly during DKD progression. At the genus level, 35 bacterial genera with continuously changing abundance along with the DKD progression were considered as key microbiota in the progression of DKD. Among them, *Oscillibacter*, *Oscillospiraceae_UCG-002*, *Megamonas*, and *Limosilactobacillus* exhibited particularly significant changes. Previous studies have confirmed that the abundance of *Oscillibacter* in DKD patients was significantly higher compared to NDKD (non-diabetic kidney disease) patients (Chen et al., 2022). Our study further demonstrated that its abundance also gradually increased during the DKD progression. The study by Chen et al. found that *Oscillibacter* intervened in host lipid homeostasis by metabolizing intestinal cholesterol, thereby reducing plasma cholesterol and triglyceride levels (Li et al., 2024). Lipid disorders are recognized as a risk factor for DKD (Li et al., 2025). Therefore, we surmised that *Oscillibacter* may exacerbate renal injury in DKD patients by interfering with lipid metabolism. Notably, previous studies have found a significant positive correlation between *Oscillospiraceae UCG-002* and total p-Cresyl sulfate (PCS) (James et al., 2025), which is also a key factor in DKD progression (Koppe et al., 2018). Our result was consistent with these findings, showing a gradual increase in abundance of *Oscillospiraceae UCG-002* with DKD progression. *Lactobacillus johnsonii*, belonging to *Limosilactobacillus*, is regarded as a beneficial bacterium. Supplementation with this bacterium can alleviate renal injury in rats with chronic kidney disease (CKD) (Miao et al., 2024). Similarly, our study demonstrated that the abundance of *Limosilactobacillus* decreased progressively during the DKD progression. Therefore, targeting *Limosilactobacillus* may be beneficial for delaying the DKD progression. Previous studies have found that *Meganomas* promoted intestinal lipid absorption by degrading inositol, thereby accelerating the progression of obesity (Wu et al., 2024). Additionally, in patients with type 1 diabetes mellitus (T1DM), myo-inositol (a type of inositol) was significantly correlated with indicators of renal injury. For instance, it showed a negative correlation with eGFR and a positive correlation with microalbuminuria (Tofte et al., 2019). This is consistent with our study. Therefore, we speculate that in DKD, the reduction in *Megamonas* may lead to an increase in inositol, which in turn exacerbates renal injury and promotes the progression of DKD. Similar to our findings, the gut microbiota with dynamically changing abundance in DKD was closely associated with the progression of DKD.

The existence of the “gut-kidney axis” regulates the DKD progression (Han et al., 2023). Previous studies on the role of gut microbiota in DKD mostly focused on metabolism and inflammation, while the role of immune cells in this process has also attracted increasing attention. The research by Yan Jia and Hui Xu’s team reported that compared with the normal control group, the proportion of CD4^+^ T cells in the DKD group is significantly increased, suggesting that CD4^+^ T cell subsets may play a role in renal interstitial injury in DKD (Jia et al., 2021). Transplantation therapy with CD4^+^ T cells has been shown to restore the homeostasis of gut microbiota, reduce the relative abundance of pro-inflammatory bacteria, and enhance the integrity of the intestinal barrier (Gómez De Las Heras et al., 2025). Therefore, the potential interaction between CD4^+^ T cells, the dynamically evolving gut microbiota in DKD, and DKD deserves in-depth exploration. Through Spearman correlation analysis, we confirmed a strong correlation between CD4^+^ T cells and the key microbiota involved in the DKD progression. We speculate that there may be some interaction mechanism between CD4^+^ T cells and specific gut microbiota, which in turn affects the DKD progression. In addition, there were significant differences in the microbiota among groups with different levels of CD4^+^ T cells, and these differences were significantly correlated with indicators related to renal function in DKD patients.

According to previous reports, effector memory (EM) CD4^+^ T cells significantly mediate the “*Oscillibacter*–Alzheimer’s disease (AD)” pathway (Zhang et al., 2024). This prompted us to investigate whether CD4^+^ T cells also play a mediating role in the “gut microbiota–DKD progression” axis. Through mediation analysis, we found that CD4^+^ T cell count can mediate the the association between gut microbiota and DKD progression, particularly within some dynamically evolving gut microbiota. Among them, *Akkermansia* showed the strongest mediating effect (Prop of Mediated = 33.025%). Our study found that the abundance of *Akkermansia* gradually increased during the DKD progression, especially significantly elevating in the late stage of DKD, and it was significantly negatively correlated with the CD4^+^ T cell count. Previous studies have revealed that *Akkermansia* maintained the homeostasis of gut microbiota and alleviated renal interstitial fibrosis (Pei et al., 2023). Transplantation of *Akkermansia muciniphila* promoted regulatory T cell (Treg) immune responses and delayed the progression of diabetes mellitus (Hänninen et al., 2018). However, emerging evidence indicates that *Akkermansia*’s protective effects are Treg-dependent, with complete abrogation of protection following Treg depletion and significant exacerbation of organ damage upon Treg clearance (Xie et al., 2025). In addition, among the 13 genera with mediating effects in this study, *Megamonas* was the only genus with negative values for both ACME and ADE. Our study found that the abundance of *Megamonas* decreased significantly with the DKD progression and was significantly positively correlated with the CD4^+^ T cell count. Previous studies confirmed that *Megamonas* can produce short-chain fatty acids (SCFAs) (Jing et al., 2023), which can significantly promote the differentiation and expansion of Treg cells, thereby exerting anti-inflammatory and immunomodulatory effects (Schwarz et al., 2021; Hu et al., 2022). Although previous studies have suggested that some of these genera may be linked to microbial metabolism or immune regulation, our study did not assess the specific mechanisms involving CD4^+^ T cell subsets. Therefore, the biological significance of these associations in DKD progression remains to be clarified.

Our study not only identified gut microbiota with continuously changing abundance during DKD progression, but also applied a mediation model to support an association among gut microbiota, CD4^+^ T cell count and DKD progression. From a new perspective, it provides a basis for future studies on immunity and gut microbiota in DKD. In addition, propensity score matching was performed in the CD4^+^ T cell subgroup analysis to reduce the influence of individual differences and enhanced the credibility of the research results. However, several limitations should be acknowledged. First, due to the cross-sectional design, causal relationships among gut microbiota, CD4^+^ T cell count, and DKD progression cannot be established. Therefore, the mediation results should be interpreted as statistical associations rather than definitive evidence of causal mediation. Second, the depth of 16S sequencing is limited, making it impossible to accurately identify down to the species level. Third, group comparisons of microbial taxa were performed using nonparametric tests on relative abundance data. This approach has inherent limitations and may be less robust than count-based or compositionality-aware methods such as ANCOM-BC. Therefore, future studies are needed to validate these findings using more appropriate statistical frameworks. Fourth, the proportion of the mediating effect of CD4^+^ T cells in the current mediation analysis was relatively low, which may be due to the small sample size. In the future, larger cohorts of DKD patients from multiple clinical centers and more immune indicators should be included. In addition, CD4^+^ T cells were assessed only by total cell count, without further characterization of their functional subsets (Th1/Th2/Th17/Treg). Given the significant functional heterogeneity of CD4^+^ T cells, this represents a limitation that constrains mechanistic interpretation. Future studies should incorporate multicolor flow cytometry and intracellular cytokine staining to characterize CD4^+^ T cell subsets, thereby clarifying their mechanistic roles in mediating DKD progression. Moreover, The CD4^+^ T cell grouping in this study could not fully eliminate residual confounding by DKD stage. Future studies with larger sample sizes are warranted to stratify CD4^+^ T cell count levels within each DKD stage to better control for the influence of DKD progression on microbiota-immune interactions. Finally, animal and cell experiments are required to validate the observed associations and further clarify the underlying biological mechanisms.

## 5 Conclusion

The gut microbiota undergoes continuous changes in abundance during DKD progression. The CD4^+^ T cell count demonstrates a mediating association in the relationship between gut microbiota and DKD progression. This study supports a potential association within the “gut–immune–kidney” axis in DKD, while the underlying immune-mediated mechanisms remain to be elucidated in future research.

## Data Availability

The datasets presented in this study can be found in online repositories. The names of the repository/repositories and accession number(s) can be found below: https://www.ncbi.nlm.nih.gov/, PRJNA881044.

## Supplementary material

Supplementary Figure S1 The composition of distinct gut microbiota and community richness across different stages of DKD. **(A)** Chao index was used to assess alpha diversity among the Early (n = 34), Middle (n = 91), and Late (n = 32) DKD groups using the Kruskal–Wallis test (p = 0.035). **(B)** NMDS calculated by Jaccard’s distances. **(C)** CPCoA was used for the comparison of inter-group differences among the three groups. The dominant bacterial taxa from the phylum to genus level were presented in the cladogram **(D)** and LEfSe diagram **(E)**. Group-wise differences in relative abundance at the phylum **(F)** and genus **(G)** levels across the three groups were assessed using the Kruskal–Wallis test. *p < 0.05, **p < 0.01, ***p < 0.001. DKD, diabetic kidney disease; Early, early stage DKD; Middle, middle stage DKD; Late, late stage DKD; NMDS, non-metric multidimensional scaling; CPCoA, constrained principal coordinate analysis; LEfSe, linear discriminate analysis effect size.

Supplementary Figure S2 Comparison of gut microbiota abundance across different levels and the correlation between key ASVs and clinical indicators. Microbial comparison among the two groups at the class **(A)**, order **(B)** or family **(C)** level through Kruskal-Wallis test. **(D)** Spearman correlation analysis between crucial clinical indicators and key ASVs when compared different DKD stages. *p < 0.05, **p < 0.01, ***p < 0.001. ASV, amplicon sequence variant.

Supplementary Figure S3 Significant differences in the richness and structure of the gut microbiota existed in the two groups with different CD4^+^ T cell levels. **(A)** Chao index was used to reflect the difference in gut microbiota richness between N_CD4^+^ T (n=44) and L_CD4^+^ T (n=22) groups using the Mann–Whitney U test (p = 0.055). **(B)** Cladogram showing the most differentially abundant taxa identified by LEfSe. Red, taxa enriched in the N_CD4^+^ T group. Blue, taxa enriched in the L_CD4^+^ T group. **(C)** Weighted UniFrac distances were used to assess significance by PCoA analysis. **(D)** PLS-DA showed the differences of gut microbiota among the two groups. Differences in relative abundance at the phylum **(E)** and genus **(F)** levels between the two groups were assessed using the Mann–Whitney U test. PCoA, principal coordinate analysis; PLS-DA, partial least squares discriminant analysis; N_CD4^+^ T, normal CD4^+^ T cell count; L_CD4^+^ T, low CD4^+^ T cell count.
