# Supplementary figures and images for: CD4+ T cell count mediates the association between gut microbiota and diabetic kidney disease progression
Source: Front Cell Infect Microbiol. 2026 May 20;16:1699989. doi: 10.3389/fcimb.2026.1699989 (PMC13216516; doi:10.3389/fcimb.2026.1699989)

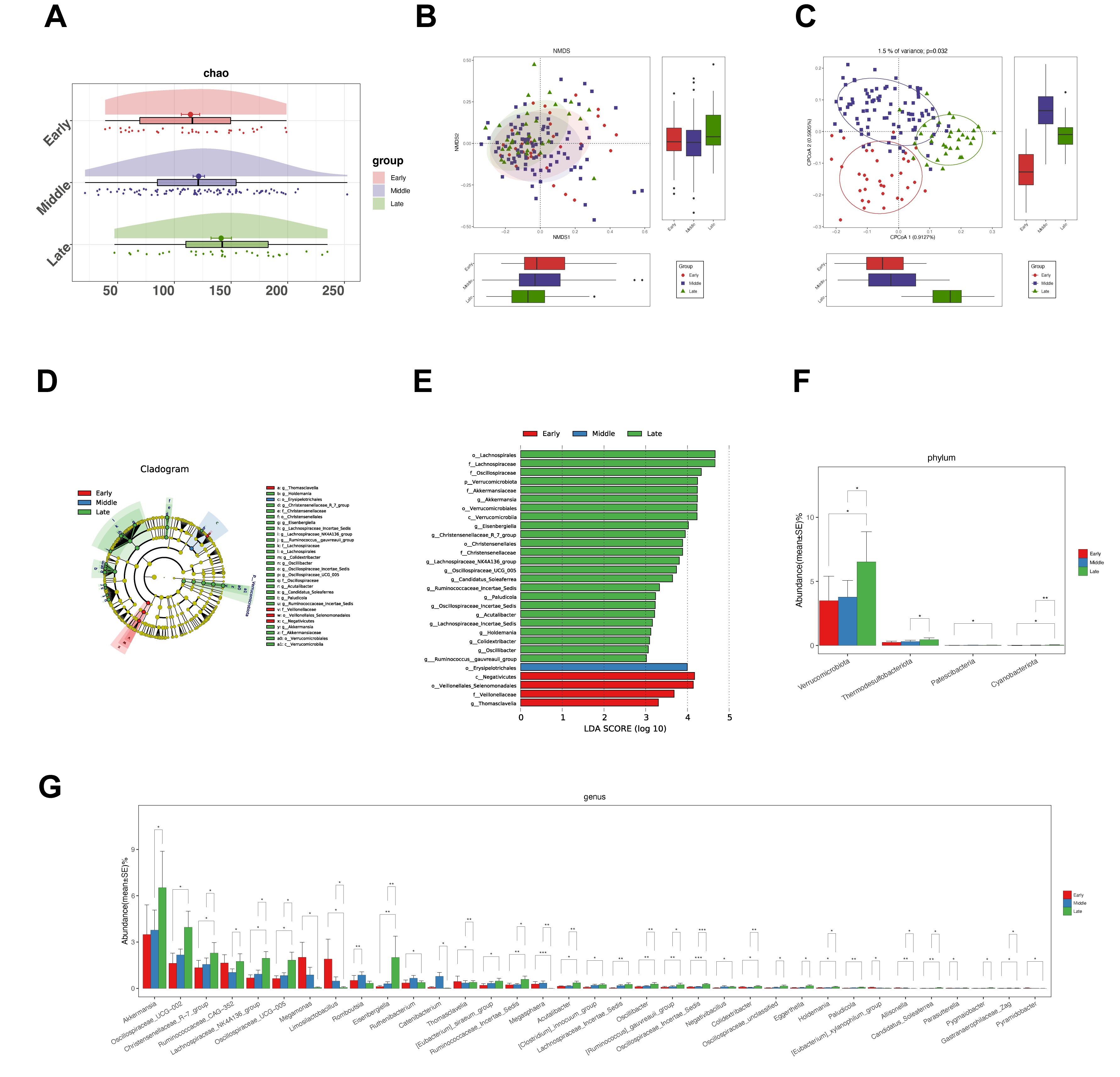

Supplement: Supplementary Figure S1 — The composition of distinct gut microbiota and community richness across different stages of DKD. (A) Chao index was used to assess alpha diversity among the Early (n = 34), Middle (n = 91), and Late (n = 32) DKD groups using the Kruskal–Wallis test (p = 0.035). (B) NMDS calculated by Jaccard’s distances. (C) CPCoA was used for the comparison of inter-group differences among the three groups. The dominant bacterial taxa from the phylum to genus level were presented in the cladogram (D) and LEfSe diagram (E). Group-wise differences in relative abundance at the phylum (F) and genus (G) levels across the three groups were assessed using the Kruskal–Wallis test. *p < 0.05, **p < 0.01, ***p < 0.001. DKD, diabetic kidney disease; Early, early stage DKD; Middle, middle stage DKD; Late, late stage DKD; NMDS, non-metric multidimensional scaling; CPCoA, constrained principal coordinate analysis; LEfSe, linear discriminate analysis effect size. [file Image1.jpeg]

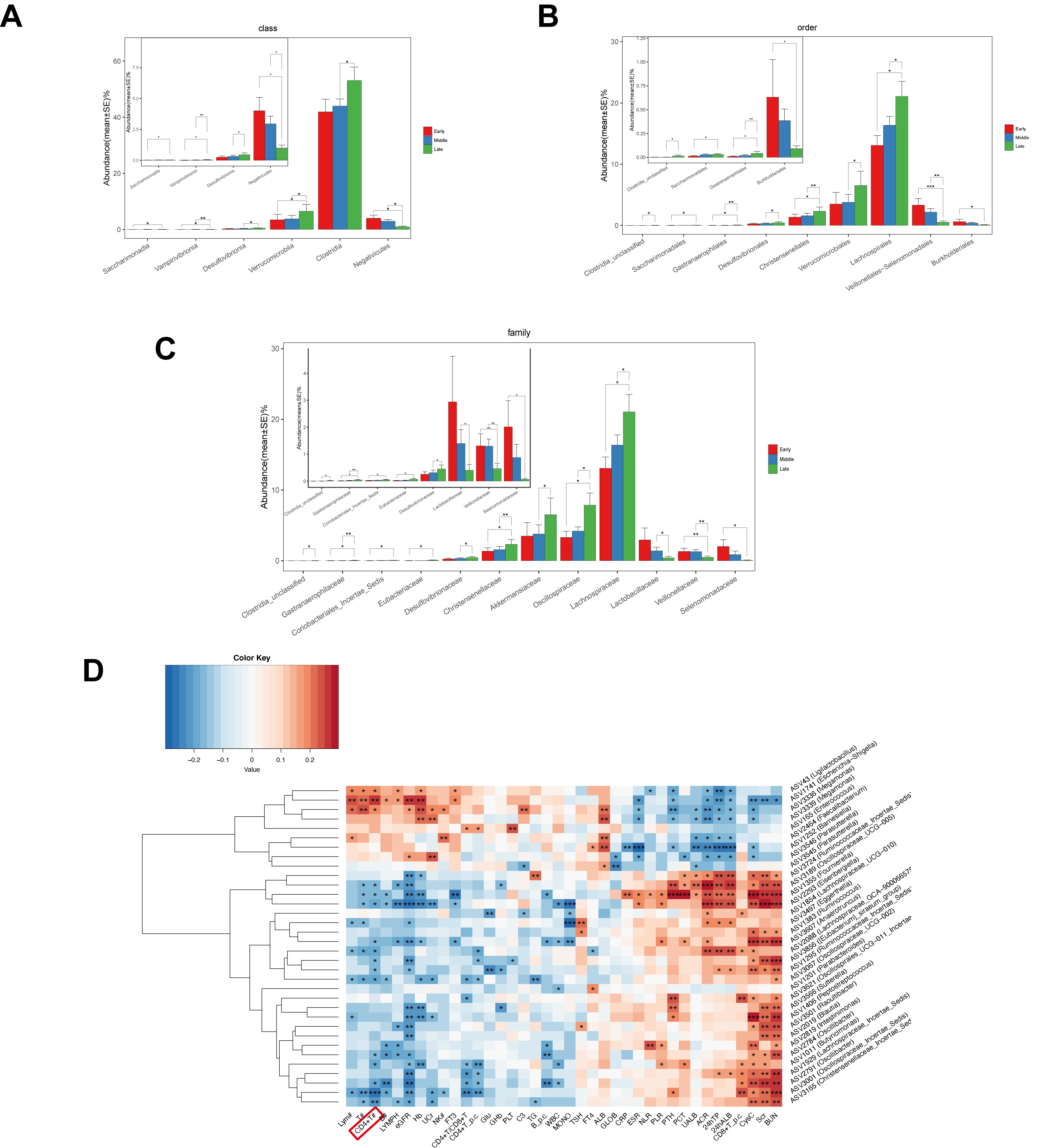

Supplement: Supplementary Figure S2 — Comparison of gut microbiota abundance across different levels and the correlation between key ASVs and clinical indicators. Microbial comparison among the two groups at the class (A), order (B) or family (C) level through Kruskal-Wallis test. (D) Spearman correlation analysis between crucial clinical indicators and key ASVs when compared different DKD stages. *p < 0.05, **p < 0.01, ***p < 0.001. ASV, amplicon sequence variant. [file Image2.jpeg]

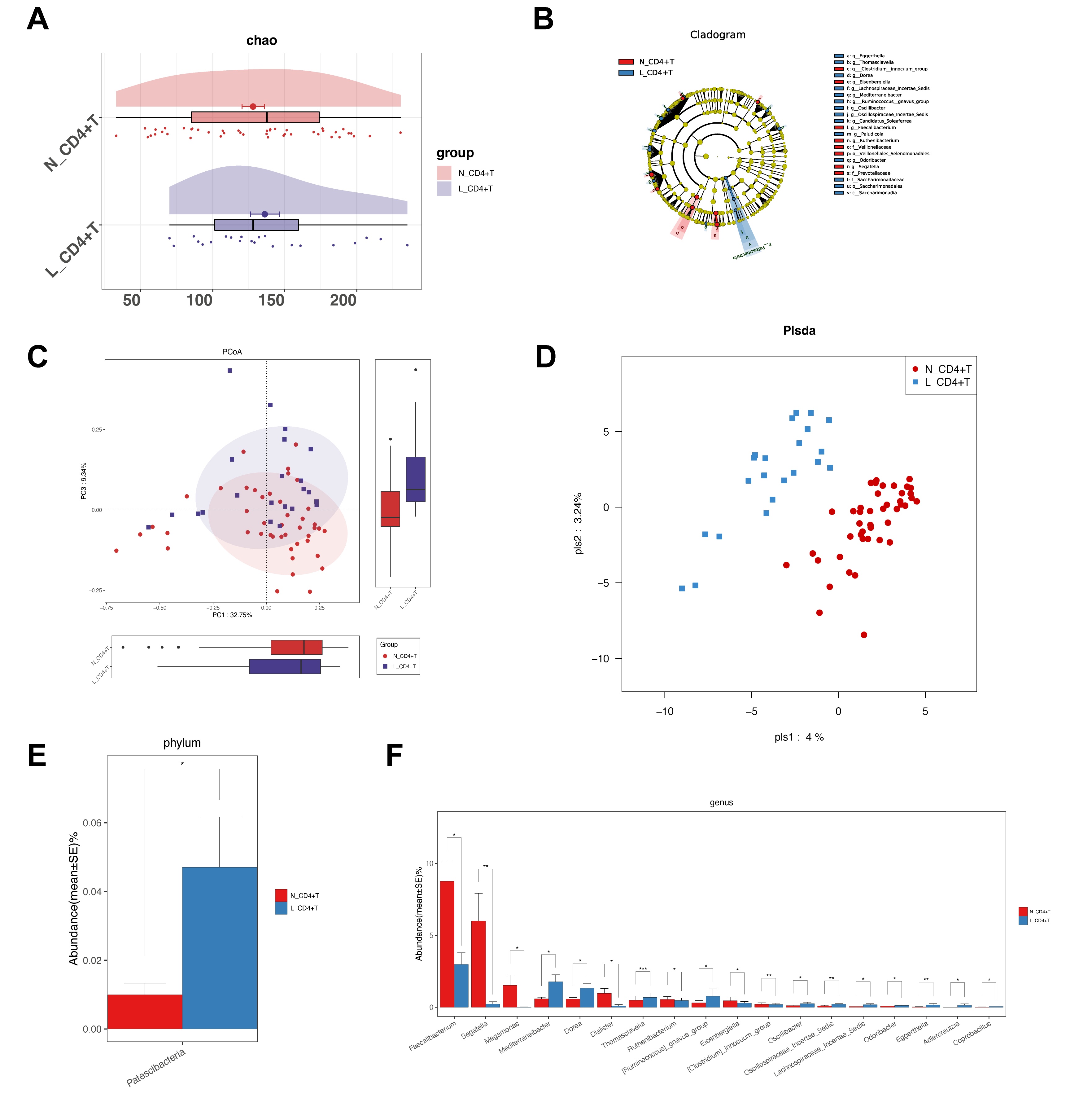

Supplement: Supplementary Figure S3 — Significant differences in the richness and structure of the gut microbiota existed in the two groups with different CD4+ T cell levels. (A) Chao index was used to reflect the difference in gut microbiota richness between N_CD4+ T (n=44) and L_CD4+ T (n=22) groups using the Mann–Whitney U test (p = 0.055). (B) Cladogram showing the most differentially abundant taxa identified by LEfSe. Red, taxa enriched in the N_CD4+ T group. Blue, taxa enriched in the L_CD4+ T group. (C) Weighted UniFrac distances were used to assess significance by PCoA analysis. (D) PLS-DA showed the differences of gut microbiota among the two groups. Differences in relative abundance at the phylum (E) and genus (F) levels between the two groups were assessed using the Mann–Whitney U test. PCoA, principal coordinate analysis; PLS-DA, partial least squares discriminant analysis; N_CD4+ T, normal CD4+ T cell count; L_CD4+ T, low CD4+ T cell count. [file Image3.jpeg]
